# Medicinal Properties of the Euphorbia Corollata

**Published:** 1833

**Authors:** 


2. *Medicinal Properties of the Euphorbia Corollata.*—This also is one of our common plants, known by the names of milk-weed, snake's milk, ipecac and Indian physic—epithets, which, however, are applied by the people to other plants of this country. It grows in cultivated fields. Dr. Zollikoffer, who is known to the profession by his researches in our Medical Botany, has published in the same Journal, a paper on its pharmaceutic and medical qualities, from which we make the following extract:

"*Pharmaceutic preparations.*—The vin. Euphorbiæ, and the pulvis corollatæ compositus. The former preparation I have made in the following way:—R. Radicis Euphorbiæ corollatæ, oz.i; vini albi hispanæ, octarius unus. dosis dr.ii. ad oz.ss.; emesis provocandi. The latter formulæ is in imitation of the pulvis ipecacuanhæ compositus, and doses not differ in the proportions, except in substituting the corollata in the place of the ipecacuanha. Both of these preparations are as prompt and effectual as remedial agents.

*Medical uses.*—This vegetable is emetic, diaphoretic, expectorant and epitastic. As an emetic it is mild and certain in its operation, rarely occasioning pain or spasms, and exciting little previous nausea or giddiness; possessing an advantage over some other remedies of this class, that when it does not prove emetic, it passes off by the bowels. The dose of the powdered root that is required to excite emesis, is from fifteen to twenty grains. This substance is better calculated to effect certain indications, than the more nauseating articles belonging to this class of remedies; and as the judicious practitioner, in the selection of an emetic, will always be guided by the nature of an indication which he intends to fulfil, if his object be to evacuate the stomach quickly and completely, he will avoid those emetics that are distinguished by their nauseating tendency, as in cases of disease which depend on a disordered state of the stomach, in connexion with undue distention, and the presence of acrid and indigestible matter: if, on the other hand, his intention be to influence some remote organ through the sympathetic powers of the stomach, an emetic of an opposite tendency may be better calculated to answer such indications, inasmuch as the nausea which they induce, greatly lessens the force of the circulation; and as the energy of absorption is generally in an increase ratio to that of the circulation, we frequently obtain from nauseating emetics considerable assistance in the treatment of different dropsical affections. The corollata is only calculated to effect the indications which are accomplished by the primary peoration of emetics, for whenever our object is to evacuate the stomach, and prevent absorption, we must take care to cut short the nauseating stage, (by such articles as produce little or none of this effect,) a precaution which is highly important in the management of cases in which poisonous substances have been taken into the stomach. My object at present is not to discuss the operation of emetics and the diversified affections in which they are not unfrequently competent to the production of the most satisfactory results, for this to me would appear to be foreign to the contemplated intention I am desirous of fulfilling by the communication of this paper. What I have said is merely intended to show the applicability of this plant to particular cases. When it is administered with the view of obtaining its diaphoretic operation, the quantity exhibited at each dose should not exceed four grains, and this proportion should be given every three hours. The pulvis corollatæ compositus may very frequently be used, particularly when a stimulant diaphoretic is indicated; for from the operation of this compound remedial agent, it would seem, that whilst the opium increases the force of the circulation, the corollata relaxes the exhalant vessels, and consequently induces a copious diaphoresis. The action of each of these articles would appear to be mutually lost, and converted into a powerful diaphoretic, thus affording a very beautiful illustration of the combination of medicines which excite different actions on the stomach and system, in consequence of which new or modified results are produced. The dose of this combination is the same of that of the Dover's powder. The expectorant operation of this plant may be procured in the dose of three grains, occasionally exhibited in a little honey, sugar and water, or any other suitable vehicle. In relation to its epispastic property, I would remark, that it possesses an advantage over the ointment prepared with the antimonium tartarizatum, in producing a beautiful display of pustules in twelve or fifteen hours after its application, which passes off in two or three days, without occasioning the least inconvenience whatever to the patient. The root in its recent state is merely to be applied contused to any part of the body, and permitted to remain a few minutes only, when a sufficient quantity of the lactescent matter will remain to produce the intended effect. A continuation of the pustular eruption may be kept up, by making an application of the contused root every forty-eight hours. It is the only vegetable substance that is entitled to attention on the account of the property it possesses of exciting an action on the surface in this way; and the only one that is calculated to answer the same purposes of the tartar emetic ointment."

